# A Hybrid-Attention Nested UNet for Nuclear Segmentation in Histopathological Images

**DOI:** 10.3389/fmolb.2021.614174

**Published:** 2021-02-17

**Authors:** Hongliang He, Chi Zhang, Jie Chen, Ruizhe Geng, Luyang Chen, Yongsheng Liang, Yanchang Lu, Jihua Wu, Yongjie Xu

**Affiliations:** ^1^ School of Electronic and Computer Engineering, Peking University, Shenzhen, China; ^2^ Peng Cheng Laboratory, Shenzhen, China; ^3^ College of Engineering, Pennsylvania State University, State College, PA, United States; ^4^ Harbin Institute of Technology, Shenzhen, China; ^5^ Beijing Normal University-Hong Kong Baptist University United International College, Zhuhai, China; ^6^ PLA Strategic Support Force Characteristic Medical Center, Beijing, China

**Keywords:** Histopathological image, Nuclear segmentation, Nested UNet, Hybrid attention, Dilated convolution

## Abstract

Nuclear segmentation of histopathological images is a crucial step in computer-aided image analysis. There are complex, diverse, dense, and even overlapping nuclei in these histopathological images, leading to a challenging task of nuclear segmentation. To overcome this challenge, this paper proposes a hybrid-attention nested UNet (Han-Net), which consists of two modules: a hybrid nested U-shaped network (H-part) and a hybrid attention block (A-part). H-part combines a nested multi-depth U-shaped network and a dense network with full resolution to capture more effective features. A-part is used to explore attention information and build correlations between different pixels. With these two modules, Han-Net extracts discriminative features, which effectively segment the boundaries of not only complex and diverse nuclei but also small and dense nuclei. The comparison in a publicly available multi-organ dataset shows that the proposed model achieves the state-of-the-art performance compared to other models.

## 1 Introduction

Histopathological imaging diagnosis is an important significance of cancer diagnosis, known as the “gold standard” of clinical tumors. Nuclear segmentation of histopathological images is a crucial step in computer-aided image analysis. Accurately segmenting the nucleus in pathological tissue sections provides powerful support for disease diagnosis, cancer staging, and postoperative treatment. However, the task of nuclear segmentation in histopathological images is still challenging, for which 1) the types of histopathological structures are complex and diverse, and there are many types and complex appearances of nuclei; 2) the nuclei are usually small and dense, leading to an overlapping challenge for nuclear segmentation.

Traditional nuclear segmentation methods have contributed to some extent, such as Otsu (Otsu, 1979), the watershed method (Yang et al., 2006), K-mean clustering (Filipczuk et al., 2011), and Grab Cut (Rother et al., 2004). However, some specific parameters or thresholds are required to set while using these methods for nuclear segmentation. Besides, the lack of generalization ability makes these methods only effective for a few types of histopathological images. With the application and development of deep learning technology in image segmentation, these traditional nuclear segmentation methods are only used as pre/post-processing steps.

In recent years, some models based on convolutional neural networks have been proposed for histopathological image analysis (Ronneberger et al., 2015), (Zhou et al., 2018), (Wollmann et al., 2019), (Qu et al., 2019). UNet (Ronneberger et al., 2015) is successfully applied to segmentation tasks in medical image analysis. The network adopts encode-decode design and has skip connections to combine low-level and high-level feature information. This framework effectively captures the contextual information of image features and has become a basic framework of the current mainstream model for segmentation tasks. However, only using the UNet framework cannot efficiently separate dense or overlapping instances. Therefore, some models have been proposed to improve the performance of UNet. Zhou et al. (2018) proposed UNet++, which reduces the semantic gap between feature maps of encoder and decoder subnets through a series of nested, dense skip pathways. Wollmann et al. (2019) proposed GRUU-Net, which integrates convolutional neural networks and gated recurrent neural networks on multiple image scales to combine the advantages of both types of networks. In addition to encode-decode architecture, dilated convolution has also been proposed and applied to segmentation tasks. Yu and Koltun first proposed a new convolutional network module specifically for dense prediction, which uses dilated convolution to systematically aggregate multi-scale context information without loss of resolution (Yu and Koltun, 2016). Qu et al. introduced the idea of dilated convolution into nuclear segmentation and proposed FullNet (Qu et al., 2019). FullNet uses several densely connected layers with different dilation factors to replace the encoding-decoding operation, thereby avoiding the loss of feature information. Experiments show that the performance of FullNet in cell nuclear segmentation is better than other comparison models. In general, although some latest models have turned their attention to the gaps of the UNet model and achieved competitive performance to some extent, they still have limitations. For example, 1) they fail to effectively identify small or dense objects; 2) they treat each pixel as a separate classification task, failing to fully consider global feature information and the relevance between different pixels.

To address the issues mentioned above, the main contributions of this paper are 1) We propose a hybrid-attention nested UNet (Han-Net), which consists of two modules: a hybrid nested U-shaped network (H-part) and hybrid attention block (A-part). 2) In H-part, we integrate a nested multi-depth U-shaped network and a dense network with full resolution to capture more effective feature information. The excellent feature extraction capability of H-part can effectively segment the boundaries of complex and diverse nuclei. 3) We propose a novel hybrid attention block (A-part) to boost attention information and explore the correlation between different pixels, thereby to effectively capture some small or dense nuclei. 4) Han-Net is compared with some recently proposed models in a multi-organ segmentation dataset. The comparison results show that the proposed model has the state-of-the-art performance.

## 2 Method

**FIGURE 1:**
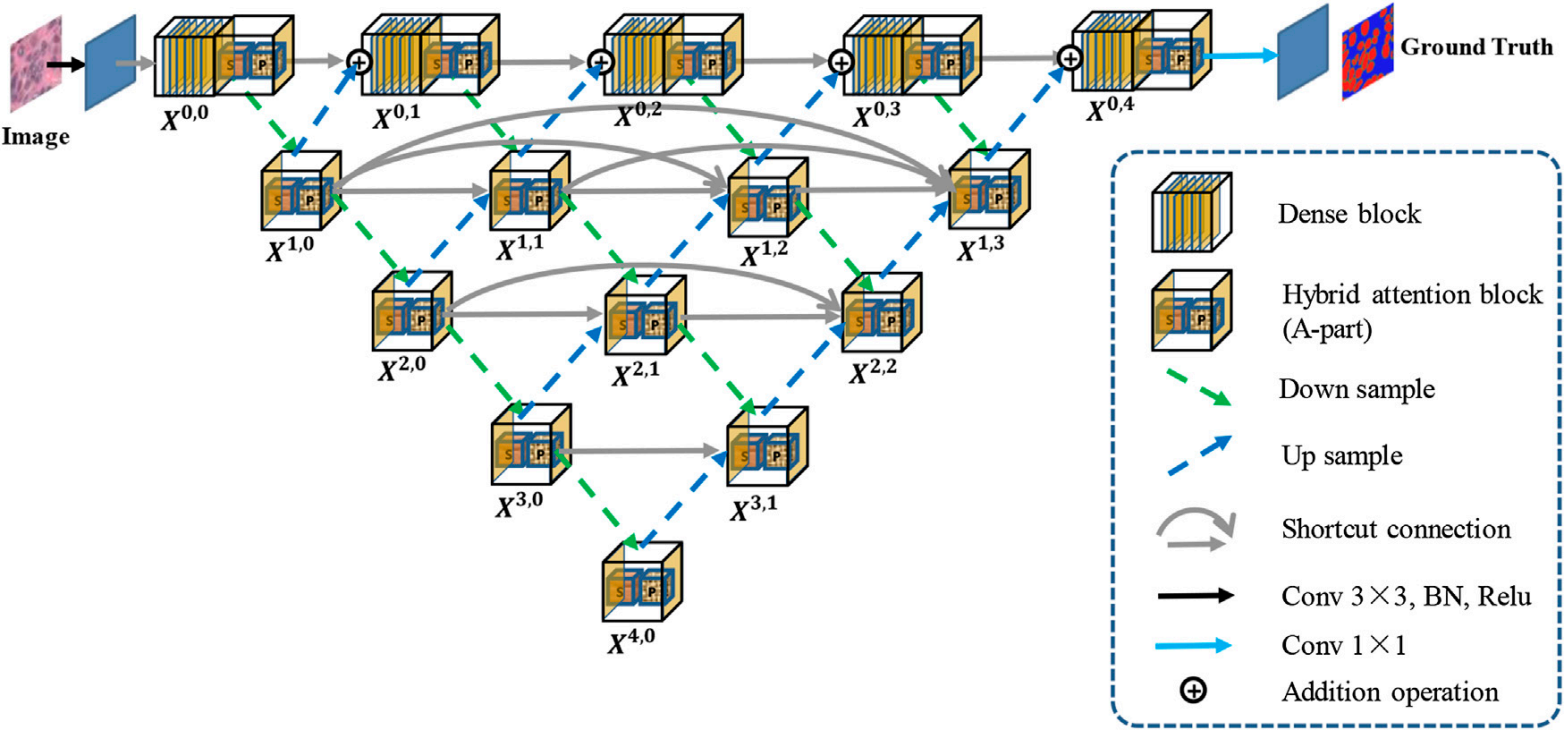
Overview of the proposed Han-Net.

Figure 1 shows an overview of the proposed Han-Net, which consists of the backbone module (H-part) and hybrid attention module (A-part). H-part is proposed to obtain multi-scale feature information and improve the capability for exploring effective features. A-part is proposed to boost attention information and capture the correlation between features. In Figure 1, each different block and process is represented by different icons and arrows. The detailed information of H-part and A-part is described in the following sub-sections.

### 2.1 Hybrid Nested U-Shaped Network (H-part)

**FIGURE 2:**
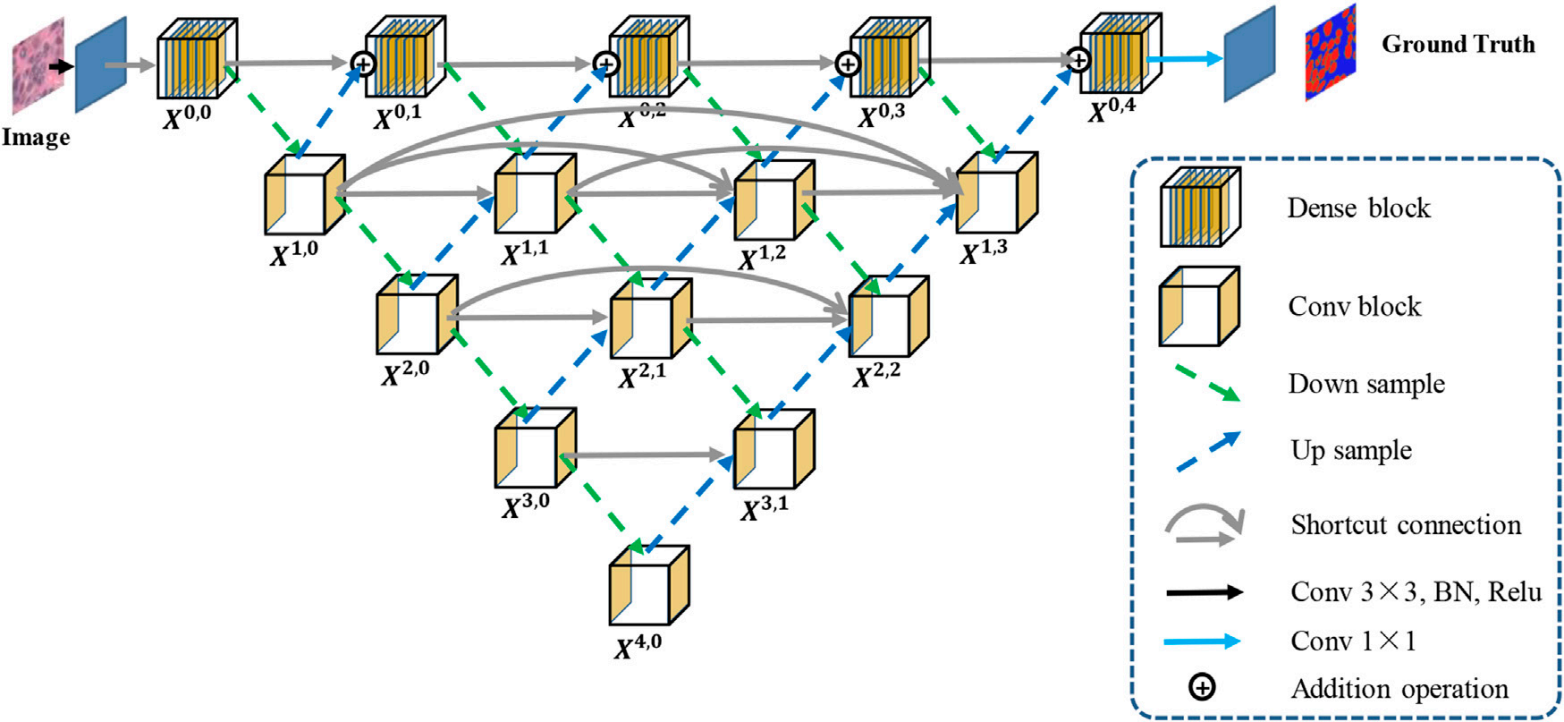
The framework of the proposed H-part.

The framework of H-part is similar to a multilayer regular triangle structure, which is composed of multiple encoders and decoders. The framework of the proposed H-part is shown in Figure 2. Inspired by UNet++ (Zhou et al., 2018), we have nested multiple conv blocks in UNet to bridge the possible semantic gap between the corresponding levels of encoder-decoder in classic UNet.

In the top layer, we added a dense network using dilated convolution to make it obtain more global feature information at full resolution. Further, we unify the channel number of the output feature map obtained from up-sampling and previous dense block, so that feature map fusion can be carried out by adding and acting as the input of next dense block. Except the top layer, we set up shortcut connections to prevent losing feature information, and use concatenate operation for the fusion of feature maps. A dense block contains $L_{n}$ convolutional layers, and a conv block contains two convolutional layers.

Moreover, we explain the calculation relationship between each block: let $x^{i,j}$ denote the output of block $X^{i,j}$, where *i* represents the index of the encoder, and *j* represents the index of dense block at the top layer. The formula for calculating the output $x^{i,j}$ of each block is shown in Eq. 1.

$$x^{i,j}=\{\begin{array}{lll} ℳ\left(A\left(x^{i,j-1}+x^{i+1,j-1}\right)\right), & & i=0,j>0\\ C\left(\left[D\left(x^{i-1,j}\right)\right]\right), & & i>0,j=0\\ C\left(\left[{\left[x^{i,k}\right]}_{k=0}^{j-1},D\left(x^{i-1,j}\right),\left(x^{i+1,j-1}\right)\right]\right) & & i>0,j>0 \end{array}$$ (1)

where ℳ(⋅) represents the convolution operation in dense block, C(⋅) represents the convolution operation in conv block, (⋅) represents the addition layer, and [⋅] represents the concatenate layer. D(⋅) and (⋅) refer to the down-sampling layer and the up-sampling layer, respectively. $x^{0,0}$ has only one input and serves as the starting position for other blocks. When j>0, each dense block has two inputs and each conv block has three or more inputs.

### 2.2 Hybrid Attention Block (A-Part)

**FIGURE 3:**
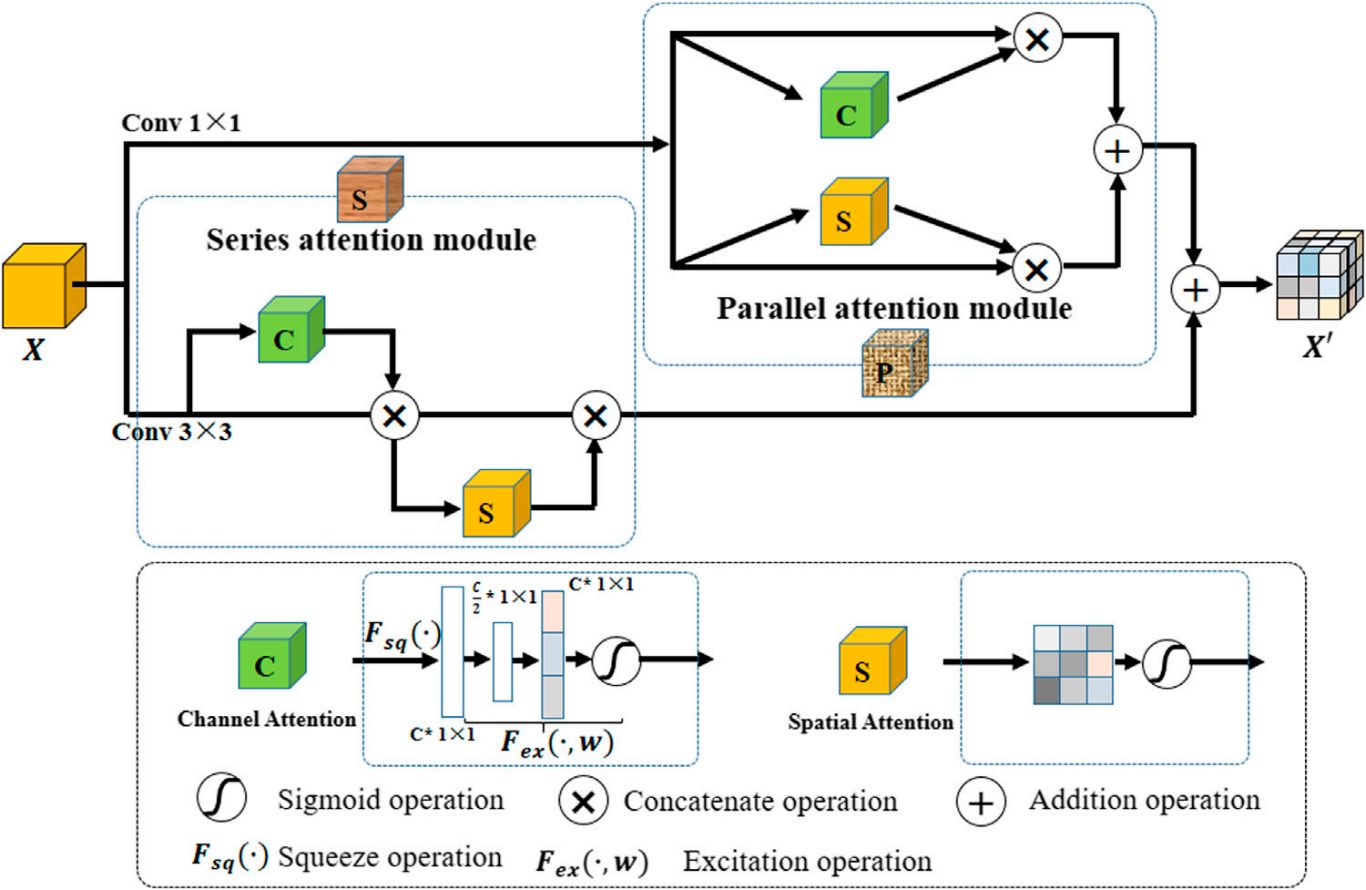
The structure of the hybrid attention block (A-part).

To highlight effective features and explore the correlation between different pixels, we propose a hybrid attention block (A-part). Its structure is shown in Figure 3. In A-part, channel attention and spatial attention constitute a series of attention module and parallel attention module through series and parallel operations, respectively. Assume that the input feature map are $X\in {\mathbb{R}}^{H\times W\times C}$. Here *H* and *W* are spatial height and width, respectively, and *C* represents the input channel. Series attention module and parallel attention module are used in convolutions of different kernel sizes, and the results obtained from these attention modules are added to obtain the output $X^{\prime }\in {\mathbb{R}}^{H\times W\times C^{\prime }}$, where $C^{\prime }$ represents the output channel.

Channel attention captures the importance of different channels in feature maps, thereby enhancing or suppressing different channels. The operation is as follows: a branch is separated after a normal convolution operation. Squeeze operation is first performed on the branch (i.e., $F_{sq}\left(\cdot \right)$ in Figure 3). It uses a global pooling operation to compress the spatial dimensions; that is, each two-dimensional feature map becomes a real number, and the number of feature channels does not change. Then, Excitation operation is performed to generate a corresponding weight for each channel through parameter *w* (i.e., $F_{ex}\left(\cdot \right)$ in Figure 3). In other words, *w* is learned to represent the correlation between feature channels.

Spatial attention aims at exploring the relative importance of each pixel on feature map. It emphasizes related spatial locations and ignores unrelated locations. The operation flow is as follows: First, a 1-channel kernel size of 1×1 convolution is performed on the input feature map, and then a sigmoid operation is performed. Then, the attention rate corresponding to each pixel is multiplied with the original feature map to obtain new feature map.

In this paper, A-part is added after each dense block in H-part to strengthen the effective information while controlling the number of channels. In the nested U-shape network of H-part, we replace the original conv block with A-part to improve extraction capability for effective feature information.

## 3 Experimental and Results

### 3.1 Dataset and Evaluation Metrics

We validate the performance of our proposed model in the Multi-Organ nuclear segmentation (MoNuSeg) dataset (Kumar et al., 2017). The dataset consists of 30 hematoxylin and eosin (H & E) stained histopathology images of size 1000×1000 from seven different organs. Following (Kumar et al., 2017), the data division mode is as follows. We extract the first three images from four organs (six images per organ) as the training set (12 images in total), the 4th image as the validation set (four images in total), and the remaining two as the Test 1 (eight images in total). six images of the remaining three organs are used as the Test 2.

In this paper, four evaluation metrics are used to comprehensively evaluate the performance of the proposed model, namely F1-score (F1), Dice coefficient (Dice), average Hausdorff distance (H), and Aggregated Jaccard Index (AJI). Among them, F1 and Dice are evaluated from pixel-level, and Hausdorff and AJI are evaluated from object-level.

### 3.2 Implementation Details

We implemented the proposed model with PyTorch 1.0. One NVIDIA Tesla V100 with CUDA 10.1 is used for computation. We treat the nuclear segmentation task as a three-class classification problem, including nucleoli, nuclear boundary, and background. During training, we use Adam as the optimizer. The initial learning rate is set as 0.001 and drop rate is set as 0.1. The dilation factors are d=(2,4,8,4,2) for the dense net in the top layer of Han-Net. The training epoch for all models in the experiment is set to be 300. In the proposed model, the number of dense layers in each dense block is 6 (i.e. $L_{n}=6$), and the number of output features maps for each layer is 16. Due to the limited size of data in the MoNuSeg dataset, we use data augmentation strategies to expand the training set to improve robustness and reduce overfitting. Data augmentations include random color transformation, random crop, horizontal flip, random elastic transformation, and random rotation transformation.

Although the model predicts three-class results during testing, we use inside class area to retrieve the boundary class area and distinguish different nuclei instances according to the boundary class area in the post-processing process. The final boundary area is obtained according to the following steps: 1) Perform dilation and erosion operations in the inside class area; 2) Subtract the area obtained by erosion from the area obtained by dilation to obtain the boundary class area.

### 3.3 Evaluation and Comparison

#### 3.3.1 Ablation Study

In the experiments, we perform several ablation studies to prove the effectiveness of the proposed method and block. We use UNet as a benchmark and compare the following strategies respectively: 1) Our nested UNet (NUNet): nested U-shaped network, that is, UNet with multiple depths, and skip connections are added to the remaining layers except for the top layer; 2) NUNet + A-part: on the basis of (1), A-part is added without dense network; 3) NUNet +Dense-top: on the basis of (1), dense network is added in the top layer; 4) Ours (Han-Net): both H-part and A-part are adopted. The performance comparison results are shown in Table 1.

**TABLE 1:** Ablation study for the Han-Net.

Method	Test 1				Test 2			
	F1	Dice	AJI	H	F1	Dice	AJI	H
UNet	0.8651	0.7987	0.5923	6.2683	0.8286	0.7701	0.5442	7.6555
NUNet	0.8765	0.7962	0.5993	6.2063	0.8422	0.7959	0.6004	6.9937
NUNet + A-part	0.8847	0.7992	0.6043	6.1121	0.8632	0.7990	0.6169	6.9149
NUNet + Dense-top	0.8777	0.7977	0.5999	6.1287	0.8688	0.8123	0.6321	6.3849
**Ours (Han-Net)**	**0.8875**	**0.8021**	**0.6091**	**6.0157**	**0.8802**	**0.8185**	**0.6419**	**5.9524**

The bold numbers show the best performance.

Comparing the performance in Table 1, some conclusions can be drawn: (1) In terms of UNet, our nested UNet performs better in the following aspects: In Test 1, F1 improves 1.14%, and other metrics also improve slightly. In Test 2, the performance of each metric gains a significant improvement. Among them, AJI shows an improvement of 5.62%. These comparison results prove that nested multi-depth U-shaped network can effectively improve segmentation performance. (2) Comparing the performance before and after using A-part, F1 achieves an improvement of 0.82%, Dice achieves 0.30%, and AJI achieves 0.50% in Test 1. In Test 2, F1 improves 2.10%, Dice improves 0.31%, and AJI improves 1.65%, respectively. The Hausdorff distance is reduced in both test sets. This shows that adding the A-part can indeed improve the segmentation performance. (3) Similarly, replacing the original conv block with dense network in the top layer effectively improves the model segmentation performance. Among them, the improvement of AJI is 3.17% in Test 2. These results prove the effectiveness of the dense network in H-part. (4) In Han-Net, which combines H-part and A-part, the segmentation performance gains more significant improvement basis of our nested UNet. Achieving F1 of 0.8875 and AJI of 0.6091 in Test 1, F1 of 0.8802, and AJI of 0.6419 in Test 2 shows an improvement of 1.10% for F1 and 0.98% for AJI in Test 1, 3.80% for F1 and 4.15% for AJI in Test 2, respectively. Dice and Hausdorff distance also have the same trend. The above results show that the several modules we proposed are effective. Further, compared with the performance improvement in Test 1, the performance improvement in Test 2 is more obvious, which reflects that after adopting the above modules, the Han-Net shows better robustness for the nuclear segmentation of different organs.

Moreover, we compare Han-Net with the novel methods proposed in previous studies. These methods include: DCAN (Chen et al., 2017), BES-Net (Oda et al., 2018), CIA-Net (Zhou et al., 2019), Spa-Net (Koohbanani et al., 2019), FullNet (Qu et al., 2019). They achieved competitive segmentation performance in the MoNuSeg dataset, respectively. Under the two evaluation metrics of F1 and AJI, the performance comparison between different methods is shown in Table 2.

**TABLE 2:** The performance comparison of different methods on the MoNuSeg dataset.

Method	Test 1		Test 2	
	F1	AJI	F1	AJI
DCAN Chen et al. (2017)	0.8265	0.6082	0.8214	0.5449
BES-Net Oda et al. (2018)	0.8118	0.5906	0.7952	0.5823
CIA-Net Zhou et al. (2019)	0.8244	**0.6129**	0.8458	0.6306
Spa-Net Koohbanani et al. (2019)	0.8281	0.6239	0.8451	0.6340
FullNet Qu et al. (2019)	0.8552	0.5946	0.8639	0.6164
Ours (Han-Net)	**0.8875**	0.6091	**0.8802**	**0.6419**

The bold numbers show the best performance.

The number of parameters and inference time are two important aspects to assess the utility of a new method. In order to further illustrate the effectiveness of the proposed Han-Net, we compared the number of parameters and inference time between Han-Net and vanilla UNet. In our experiment, Han-Net and vanilla UNet have same number of channels in each feature layer Xi (i.e. Xi, i = {0, 1, 2, 3, 4}). The channel numbers in each feature layer Xi are 64, 128, 256, 512, and 1024 respectively. The experimental results show that the parameters of Han-Net and vanilla UNet are 31.04 MB and 30.58 MB, respectively. In the experiment, we replaced the original ‘TransposeConv’ upsampling approach in UNet with ‘bilinear interpolation’ to reduce the parameters. In other words, the increase in parameters brought by the proposed modules is close to the decrease in parameters brought by the above operation. On the other hand, the experimental results show that the inference time required by Han-Net and vanilla UNet is basically the same, which means that Han-Net does not require additional inference time. These two comparative experiments also reflect the effectiveness of our proposed Han-Net.

From the performance comparison in Table 2, our proposed Han-Net achieves the state-of-the-art performance under F1 in Test 1, Test 2, and AJI in Test 2. It achieves 0.8875 of F1 in Test 1, 0.8802 of F1 and 0.6419 of AJI in Test 2 respectively, which shows an improvement of 3.23% for F1 in Test 1, 1.63% of F1 and 0.79% of AJI in Test 2. The performance of Han-Net’s AJI in Test 1 is almost the same as the optimal CIA-Net. Therefore, Han-Net can be considered to reach the state-of-the-art performance in MoNuSeg dataset.

#### 3.3.2 Qualitative analysis

**FIGURE 4:**
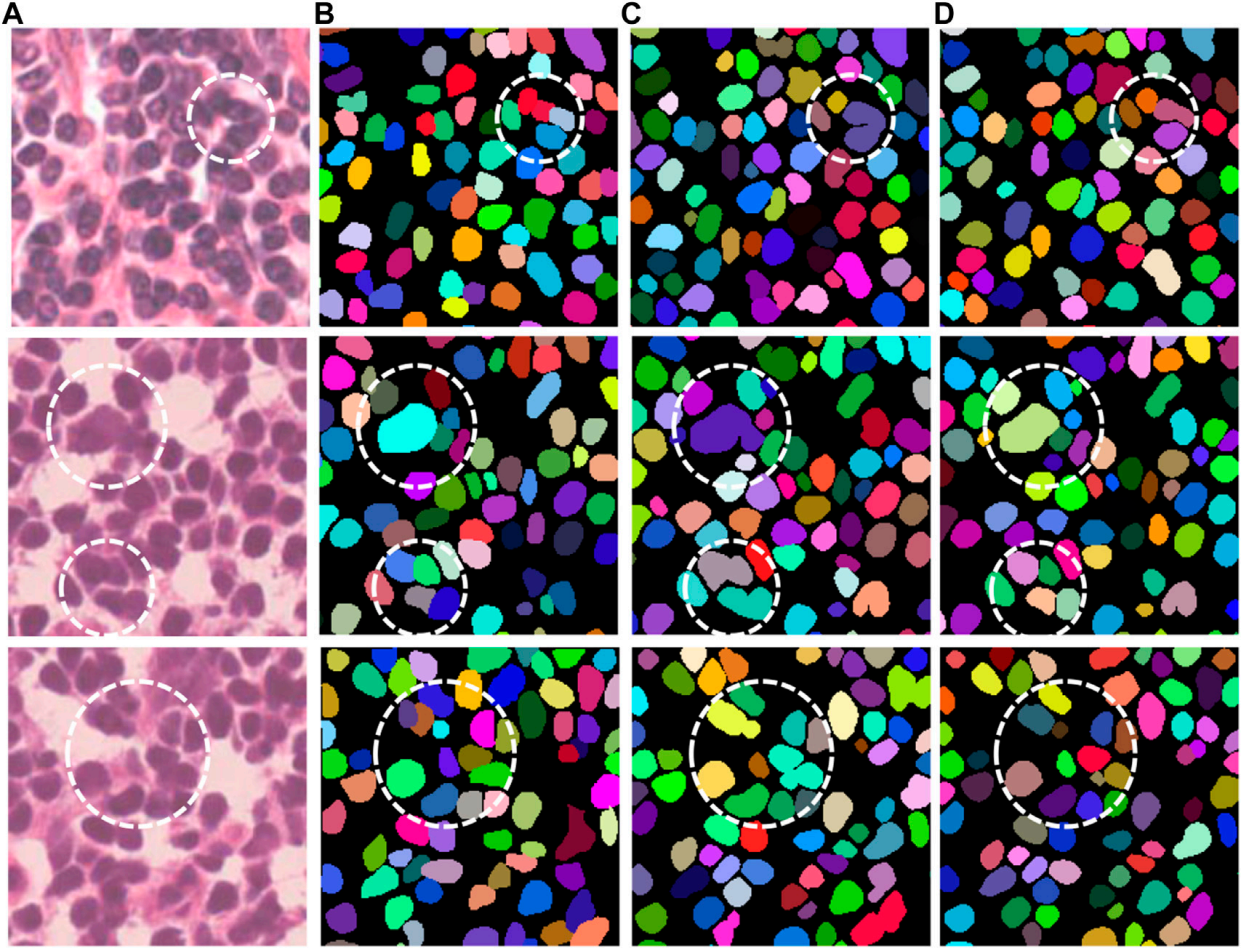
Segmentation results of UNet and Han-Net in MoNuSeg dataset.

Figure 4 shows several representative examples with challenging cases from the MoNuSeg dataset, which includes nuclei with irregular and densely distributed nuclei. That is, the relevant cases in Figure 4 are shown by a white dotted circle. It can be obtained from these images that compared with UNet, our proposed Han-Net achieves better segmentation results in some challenging regions.

## 4 Discussion

In this paper, we propose a hybrid nested attention UNet (Han-Net) for nuclear segmentation in histopathological images, which consists of H-part and A-part. Among them, H-part combines nested U-shaped network and dense network with full-resolution to obtain more effective multi-scale feature information. The A-part is proposed to enhance the effective features and suppress the invalid features, so that the proposed model can learn the morphological information of the nuclei. The experiment results prove that the Han-Net achieves state-of-the-art segmentation performance in the MoNuSeg dataset. In future work, we will consider pruning Han-Net to make it a lightweight network, and try to integrate other methods to decouple the boundary and inside.

## Data Availability

The original contributions presented in the study are included in the article/Supplementary Material, further inquiries can be directed to the corresponding authors.
